# Life-threatening complication of retropharyngeal abscess in an infant: a case report

**DOI:** 10.1186/s13256-023-04101-x

**Published:** 2023-08-27

**Authors:** Ayaz Ur Rehman, Salman Khan, Awais Abbas, Hamdan Ahmad Pasha, Qalab Abbas, Naveed Ur Rehman Siddiqui

**Affiliations:** 1 Department of Pediatric and Child Health, Aga Khan University Hospital, Karachi, Pakistan; 2 Department of ENT, Aga Khan University Hospital, Karachi, Pakistan

**Keywords:** Retropharyngeal abscess, Deep neck space infection, Airway obstruction, Cervical lymphadenopathy

## Abstract

**Background:**

Deep neck space infections are uncommon in infants. Retropharyngeal abscess (RPA) is a deep space neck infection that can present with subtle signs and symptoms. Delay in diagnosis can lead to life-threatening complications. Here we describe life-threatening complication of retropharyngeal abscess.

**Case presentation:**

We report a life-threatening complication of retropharyngeal abscess in 10-month old Asian infant weighing 8.2 kg. The patient presented with fever, right-sided neck swelling, hoarseness of voice, and respiratory distress. The clinical and radiological findings were suggestive of airway obstruction complicated by retropharyngeal abscess. The patient was urgently taken to the operating room and underwent (grade 3) intubation. After stabilization, the patient underwent endoscopic trans-oral incision and drainage, during which 5 cc pus was aspirated. Antibiotics were prescribed for 2 weeks following the procedure based on the sensitivity result of the pus culture.

**Conclusion:**

In retropharyngeal abscess, a delay in diagnosis can result in life-threatening complications. This report highlights the importance of prompt recognition of a threatened airway and the management of retropharyngeal abscess by emergency physicians. If an emergency physician suspects RPA with airway obstructions, the airway should be immediately secured in a secure environment by otolaryngologist and an anesthesiologist.

## Background

Retropharyngeal abscess (RPA) is defined as an abscess in the space between the buccopharyngeal fascia and the prevertebral fascia [1]. It is more common in children under the age of 5 years and has winter-spring seasonality [2]. Upper respiratory tract infection is the most common cause of deep neck infections in children, followed by odontogenic origin, as compared to foreign body such as fish bones and trauma is being the most common cause in adults [3, 4]. Early diagnosis and management can prevent life-threatening complications such as airway obstructions, mediastinitis, and tracheitis. We report the case of 10-month old infant with airway obstruction complicated by retropharyngeal abscess. In this case report, we discuss timely recognition of threatened airway and management of retropharyngeal abscess.

## Case presentation

A previously healthy 10-month-old Asian infant (body weight 8.2 kg), was referred to us from secondary care with complaints of fever, right-sided neck swelling for ten days, and hoarseness of voice with respiratory distress for 3 days. The patient household had satisfactory socioeconomic, and their parents were not consanguineous, living with siblings in the same residence. The infant received exclusively breast milk until 6 months of age, at which time a weaning diet was initiated. At the time of presentation, the infant was still on a weaning diet.

The child's clinical examination revealed fever with a temperature of 39.2 degrees Celsius, tachycardia with a heart rate of 176 beats per minute, a blood pressure of 114/59 mmHg and a respiratory rate of 45 breaths per minute. The neck examination revealed bilateral swelling more prominent at the right lateral aspect of the neck. Systemic examination showed increased work of breathing with resting stridor, indicative of airway obstruction. The rest of systemic examinations was unremarkable.

**Fig. 1:**
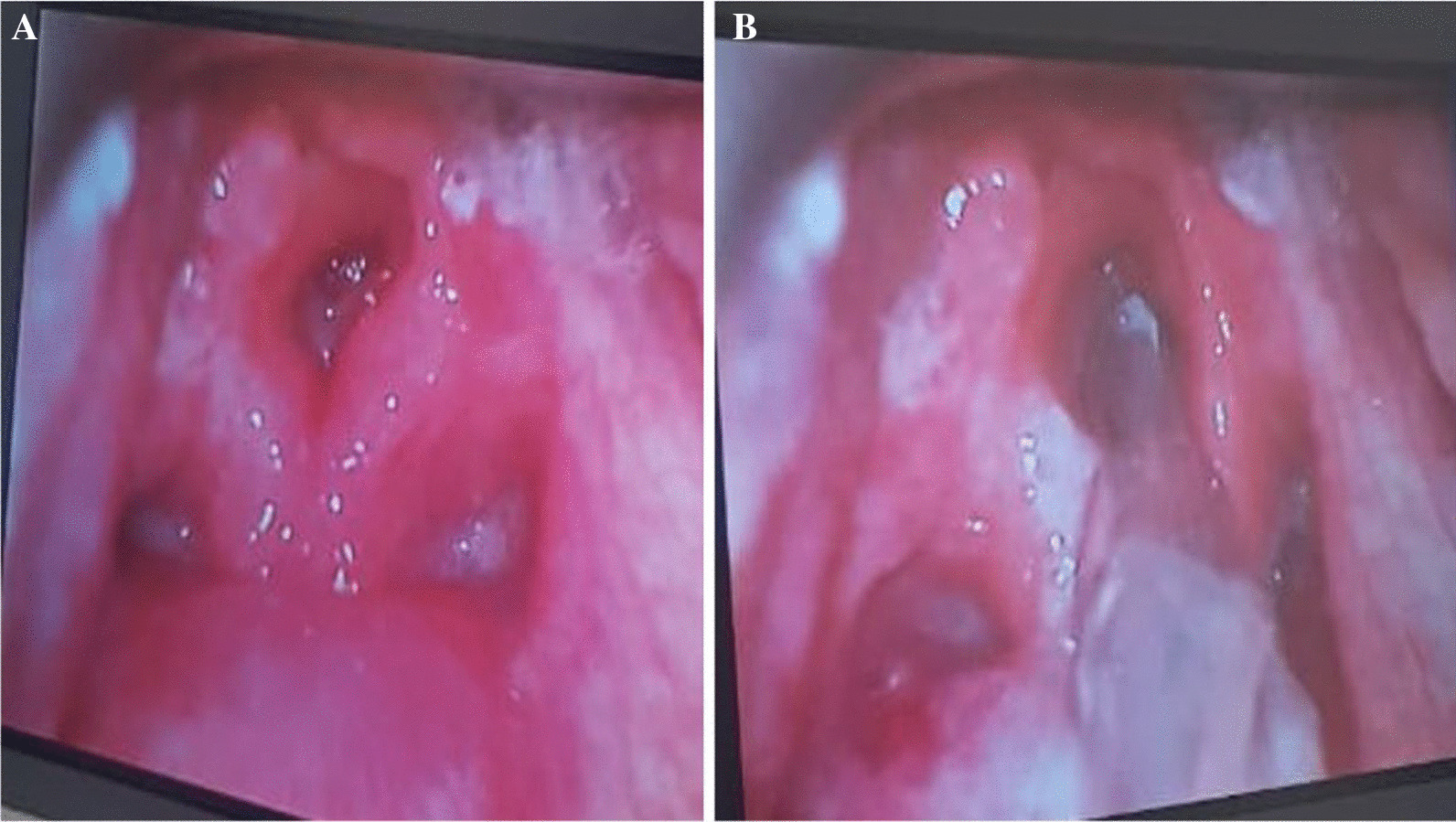
**A** Video laryngoscopy showing erythema redness and swelling around epiglottis, aryepiglottic fold and surrounding laryngeal mucosa. **B** Interval placement of endotracheal tube

**Fig. 2:**
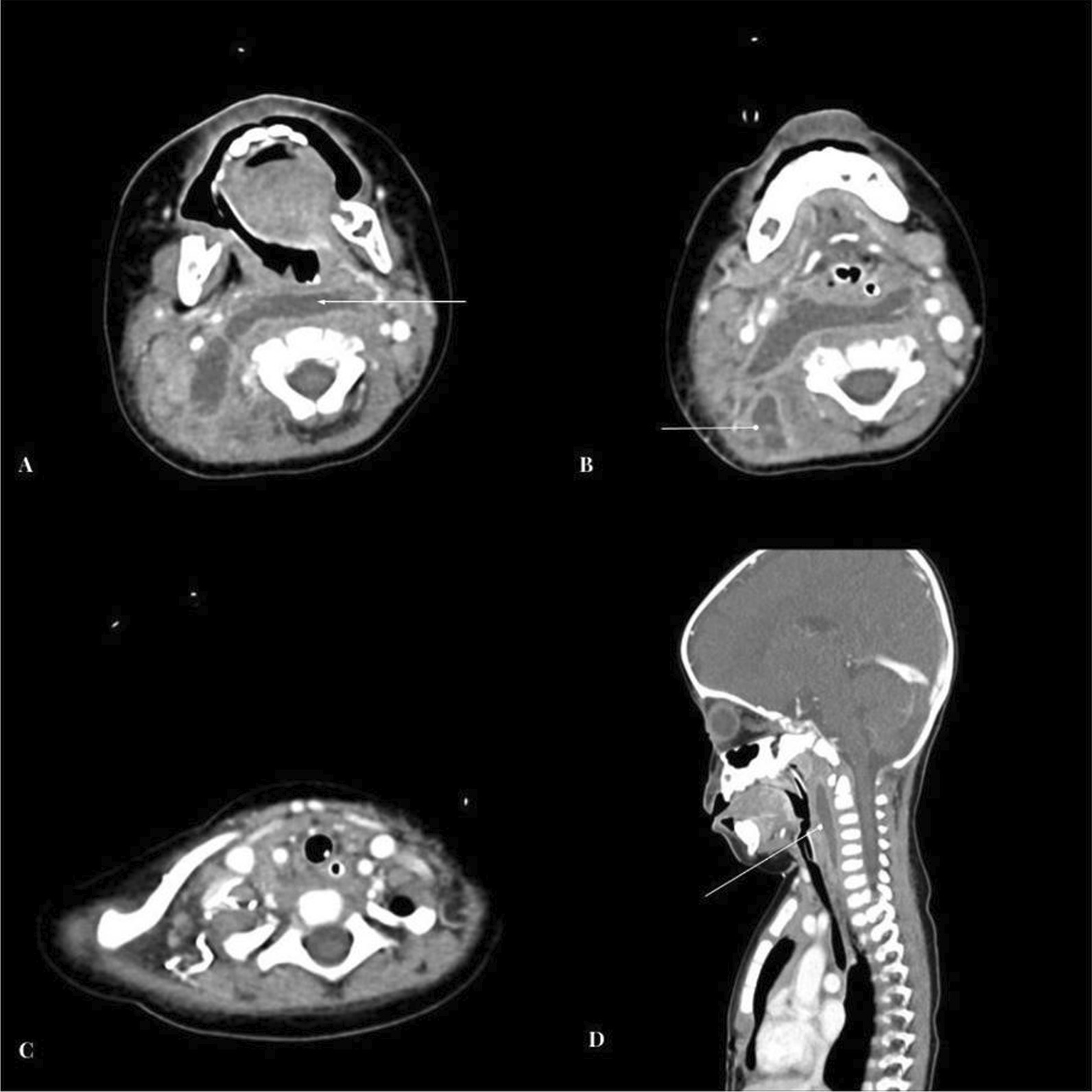
Contrast enhanced CT images through the neck. **A** Peripherally enhancing collection is identified in the retropharyngeal region, with extension along the paravertebral muscles on right side into the right posterior neck suggestive of retropharyngeal abscess formation (white arrowhead). **B** Additionally loculi of abscesses are identified within the muscle planes of right posterior neck. **C** Image at the level of superior mediastinum shows no evidence of extension of abscess into the mediastinum. **D** Midline sagittal image shows the inferior extent of the lesion to be terminating in hypopharynx

Ultrasound neck examination revealed bilateral enlarged cervical lymph nodes of varying sizes, with the largest on the right side 15 mm*10 mm and 12 mm*7.9 mm on the left side. Laboratory testing revealed leukocytosis with a white blood cell count of 30.2 k/L and a left shift. Blood analysis revealed 53% neutrophils, 0.3% eosinophils, 6.3% monocytes, 39.2% lymphocytes, 9.4 g/dL hemoglobin, 1113 k/L platelets, 72.5 mg/L C-reactive protein, and pH 7.43, 30.3 mmHg partial pressures of carbon dioxide and 211 mmHg partial pressures of oxygen, 19.8 meq/L bicarbonate, and − 3.5 meq/L base excess. The first dose of intravenous antibiotic Ceftriaxone at a dose of 65 mg/kg/day was administered. The patient was urgently taken to the operating room, where the child intubated for airway protection by an on-call anesthesia consultant. The intubation was grade 3 and was done with video laryngoscopy, as shown in Fig. 1A and B. Computed tomography of the neck showed a large rim-enhancing fluid collection in the retropharyngeal space with extension in to right paravertebral space with surrounding inflammatory changes, cervical lymphadenopathy, as shown in Fig. 2. The patient was mechanically ventilated, intubated, and admitted to the Pediatric Intensive care unit. Broad-spectrum antibiotics, including intravenous ceftriaxone (65 mg/kg/dose once daily), vancomycin (15 mg/kg/dose every eight hourly) and metronidazole (10 mg/kg/dose every eight hourly) were administered. Otolaryngology was consulted urgently, and endoscopic trans-oral incision and drainage were performed, during which 5 cc pus was aspirated and it was immediately transported to the laboratory. Gram stain of Pus was negative. Pus culture revealed enterococci, Candida albicans, while tracheal culture and Gram stain for klebs-loffler were negative. The antibiotic was deescalated to Amoxicillin + clavulanic acid (30 mg/kg/dose every eight hourly), and oral fluconazole (10 mg/kg/dose once daily) was started for Candida albicans. Blood cultures were taken on two occasions, but both results were negative for aerobes and anaerobes. Post-procedure, the patient had an uneventful PICU course, was extubated on the second postoperative day. During the recovery phase, he was kept NPO initially and then he received nasogastric feeding, with breast-feeding resuming on the fourth postoperative day. The progression of feeding was based on patient clinical condition, which was well tolerated. Antibiotics were administered to the patient for a total of 2 weeks along with ten days of antifungal treatment. Follow-up appointments with the infectious disease and surgical team were scheduled to monitor progress and address any active issues, and the patient was discharged in stable condition on the fifth postoperative day. The patient returned to our hospital 1 week after discharge for follow-up appointment, during which their condition was reassessed and it was observed that patient health had improved since their previous visit.

## Discussions

This case report describes a life-threatening complication of retropharyngeal abscess (RPA) in a 10-month-old Asian infant presenting with fever, right-sided neck swelling, hoarseness of voice, and respiratory distress. A part from existing literature, we highlight the development of rare complication such as airway obstruction by RPA, and prompt multidisciplinary management in securing the airway and successfully treating the abscess.

Sign and symptoms of retropharyngeal abscess are similar to upper respiratory tract infections. Fever, sore throat, and torticollis are early symptoms of deep space neck infection, whereas late symptoms include respiratory distress, hoarseness, neck stiffness, and stridor [5]. Delay in diagnosis can lead to life-threatening complications, such as airway obstruction, mediastinitis, cavernous sinus thrombosis, and rupture of abscess in the trachea or pharynx. Retropharyngeal abscess is polymicrobial caused by both aerobes and anaerobes; therefore, it requires antibiotics that are effective against all potential pathogens [6].

The most common organisms that are usually associated with deep neck infections are *Staphylococcus aureus*, *S. viridans*, *Klebsiella pneumonia*, Bacteroides, and Peptostreptococcus [7].

In symptomatic patients, computed tomography (CT) scan is highly sensitive (92%) in diagnosing abscess [8]. The CT scan determines the extent of abscess and involvement of adjacent structures. CT neck findings, coupled with clinical symptoms are helpful in surgical planning. However, in a well-child with retropharyngeal abscess, positive findings of lateral cervical X-ray coupled with clinical symptoms should suffice in diagnosing and managing the abscess conservatively [8].

Treatment options for retropharyngeal abscess range from a prolonged course of intravenous antibiotics to surgical drainage. Surgery is indicated in patients with a retropharyngeal abscess greater than 2.5 cm on CT scan, as in our case, RPA with complications, and patients with small RPA who are not responding to empiric antibiotics up to 48 h. The surgical approach depends on the site of the abscess. The external approach is preferred for uncomplicated cases, such as superficial abscess, and patients in whom adjacent vital structures are displaced medially, as on CT scan findings [7]. Intraoral approaches is superior in selected cases and have the advantage of short hospital stays, less morbidity, lower hospital cost and avoiding neurovascular injuries, as in our case. Patients with RPA less than 2.5 cm and non-symptomatic RPA should be conservatively managed with empiric antibiotics. Empiric intravenous antibiotics should be started as soon as possible, and the choice of empiric antibiotics should include penicillin combination with beta-lactamase inhibitor (Clavulanic acid and ampicillin-sulbactam) or beta-lactamase-resistant (Cefoxitin, Cefuroxime, imipenem, and meropenem), coupled with antianerobic antibiotics (metronidazole, clindamycin). The incidence of community-acquired methicillin resistant staphylococcus aureus is increasing in deep neck space infections [9, 10]. The adjustment of antibiotic should be done as per the culture report and clinical response of patient. In our case report, we started with ceftriaxone, vancomycin, and metronidazole and then deescalated to oral Augmentin and fluconazole, as per culture report. Intravenous antibiotics should continue until the patient shows improvement and becomes afebrile [11].

A small amount of edema in the airway can cause significant airway obstruction in infants. Intubation is very difficult in patients who present with airway obstruction secondary to deep neck space infection. Direct laryngoscopy and multiple unsuccessful attempts can puncture the abscess and cause aspiration of pus, which can lead to pneumonitis, tracheitis, and bronchitis. Patient with suspected RPA presenting with signs of airway obstruction, such as tripod or sniffing position, cyanosis, unable to speak, and tachypnea with grunting, should involve an anesthesiologist and otolaryngologist in promptly securing airway in a controlled setting, as in our case.

## Conclusion

Delay in the diagnosis of retropharyngeal abscess can lead to life-threatening complications. The management of RPA is multidisciplinary and involves various teams, including intensivists, otolaryngologists, anesthesiologists, and infectious disease specialists. If emergency physician suspect RPA with airway obstructions, the airway should be promptly secured by otolaryngologist and anesthesiologist in a secure environment. Once the airway is secured, a CT scan with contrast should be performed. Clinical symptoms coupled with CT scan neck finding can guide regarding surgical or conservative management.

## Strength and limitation of our case report

This case report present a rare and life-threatening complication of retropharyngeal abscess with airway obstruction, and highlights the importance of multidisciplinary team approach in providing timely and lifesaving intervention in such cases.

Management of such cases will be challenging especially in resource limited setting where the expertise are limited in maintenance of emergent airway, so timely and early referral of these patients would help to adequate facility would make an impact.

## Data Availability

The datasets during the current study are available from the corresponding author on reasonable request.

## Abbreviations

RPA: Retropharyngeal abscess
ER: Emergency room
OR: Operating room
CT: Computed tomography
NPO: Nil per oral
